# "Girl Power!": The Relationship between Women's Autonomy and Children's Immunization Coverage in Ethiopia

**DOI:** 10.1186/s41043-015-0028-7

**Published:** 2015-09-18

**Authors:** Jane O. Ebot

**Affiliations:** Service Delivery Improvement Division, Office of Population and Reproductive Health, Bureau for Global Health, United States Agency for International Development (USAID), 2100 Crystal Drive, Arlington, VA 22202 USA

**Keywords:** Immunization, Household autonomy and health, Ethiopia, Children

## Abstract

**Background:**

Although immunizations are efficient and cost effective methods of reducing child mortality, worldwide, approximately 2 million children die yearly of vaccine-preventable diseases. Researchers and health organizations have detailed information on the positive relationship between women’s autonomy and children’s health outcomes in developing countries.

**Methods:**

This study investigates the links between women’s household autonomy and children’s immunization status using data from a nationally representative sample of children aged 12–30 months (*N* = 2941) from the 2011 Ethiopia Demographic and Health Survey.

**Results:**

The results showed that women’s socioeconomic status and household autonomy were significantly associated with children’s immunization status.

**Conclusion:**

Overall, the implications of this study align with those of the Millennium Development Goal #3: improvements in women’s household autonomy are linked to more positive child health outcomes.

## Background

Reducing child mortality rates in Sub-Saharan Africa (SSA) has been one of the leading focuses of the Millennium Development Goals (MDG). The target of MDG 4 focuses on reducing child mortality rates by two thirds by 2015 [1]. As a way to achieve country-specific MDG 4 targets, SSA countries have created national immunization programs as efficient and cost effective ways to reduce child mortality and the number of children with life-threatening infectious diseases. The World Health Organization (WHO) recommends children be fully immunized by age 1 to prevent most of the common childhood diseases. Fully immunized children receive one dose of tuberculosis (BCG); three doses of diphtheria, pertussis, and tetanus (DPT) and polio; and one dose of measles [2]. In 2012, an estimated 2,000,000–3,000,000 deaths were averted by vaccinations, yet approximately 19,000,000 children were not fully immunized, 16,000,000 of whom resided in eight SSA countries [2].

Health organizations have stressed the importance of maternal decision-making in improving children’s health outcomes in developing countries. During the 1994 Cairo International Conference on Population and Development, policymakers from around the world called for greater focus on improving women’s autonomy to address health needs in developing countries [1]. MDG 3 promotes gender equality and women’s autonomy as important means of improving women’s and children’s health outcomes [3]. Yet limited and uneven progress from the MDG 3 has necessitated more precise research detailing the importance of women’s autonomy. Therefore, the aim of this analysis is to investigate the association between Ethiopian women’s household autonomy and children’s immunization status, which is important for both theoretical and policy reasons.

Ethiopia is the setting for this analysis because it is characterized by both low levels of full immunization coverage and women’s autonomy. Ethiopia, SSA’s second poorest country based on gross domestic product [4], is one of the few African countries that is expected to reach its country-specific MDG 4: between 1990 and 2010, under-five mortality rates declined from 167 to 88 deaths per 1000 children, an average annual decline of 6.3 % [5]. Among the many government-sponsored health sector initiatives, the increase of immunization coverage has been a key method toward successfully meeting MDG 4. As of 2010, progress in immunization coverage against diphtheria, pertussis, tetanus, and measles increased, though more than 75 % of Ethiopian children were still not fully vaccinated [6]. Given these low levels of immunizations, it is critical to research ways to increase their coverage.

Many studies have linked greater women’s household autonomy and reductions in infant mortality [7] and better child nutritional outcomes [8, 9]. Decision-making, or autonomy, is the capacity to manipulate and have control over one’s personal environment, in order to make decisions about one’s own livelihood or those of family members [10]. More autonomous women have increased decision-making abilities concerning marriage candidates [11], contraception [12], sexual activity [13], and access to health care and resources [14]. More autonomous women can also improve their children’s health compared to less autonomous women. As children’s primary caregivers, women are typically the first to recognize symptoms in sick children and invest time and income to improve their children’s health and nutrition [15]. Even before children are born, more autonomous women would have more access to safe delivery services and antenatal care, which would influence the knowledge and access they have in relation to vaccine campaigns and information [10]. Yet male heads of household most often make health care and household financial decisions in traditional societies [15]. Women’s limited household autonomy may draw limited benefits for their children from available access to child health care. For example, Woldemicael and Tenkorang analyzed the association between women’s autonomy and health-seeking behavior in Ethiopia and the results showed that more autonomous Ethiopian women were more likely to access health care compared to Ethiopian women with little household autonomy [14]. Woldemicael [16] used Demographic Health Surveys (DHS) data from Ethiopia and Eritrea to show that net of socioeconomic status, sole decision-making about visits to family/relatives increased the use of antenatal care in both countries, though sole decision-making about household purchases increased antenatal care only in Eritrea. Therefore, women’s household autonomy becomes a means through which women can use health services to reduce their young children’s risks of mortality and morbidity.

Early marriages, high fertility rates, and low levels of educational attainment, coupled with deep-rooted patriarchal beliefs regarding the role and status of women, have negatively affected Ethiopian women’s autonomy. By age 16, most Ethiopian women leave their families and communities for their new husband’s village or home [6]. Women who marry early typically have less mobility and autonomy than unmarried women or women who did not marry early and are at greater risk of intimate partner violence [17–19]. Additionally, Ethiopian women have high adolescent birth and total fertility rates which are negatively associated with children’s health and care: the 2010 adolescent birth rate was 109 births per 1000 women, the 15th highest in the world, and the current total fertility rate is 5.39 births per woman [19, 20, 21].

Although researchers have found clear relationships between women’s autonomy and health outcomes, certain limitations necessitate further study. First, few studies have focused on the relationship between women’s household autonomy and children’s immunization status, despite its recognized importance improving children’s health and odds of survival [22]. Second, prior to the inclusion of questions on women’s household status in the DHS and the Multiple Indicator Cluster Surveys (MICS), many scholars used women’s education as a proxy measure of women’s autonomy. Educational attainment is a pathway through which women can improve their livelihoods and children’s health outcomes [23], yet it does not take into account other means through which women can gain autonomy, especially for those who have not received any formal education: approximately 50 % of Ethiopian women aged 15–49 years of age had not received any formal education as of 2011 [6].

This study builds on work by Singh et al. [22] and Singh et al. [24] by including both a household decision-making index and individual-level measure of women’s household autonomy from the 2011 Ethiopia Demographic and Health Survey (EDHS) empowerment and gender modules [22, 24]. This method allows for an overall understanding of women’s household autonomy and also highlights the importance of each separate measure of decision-making within the composite household measure. For example, increased decision-making abilities concerning visits to family/relatives may improve children’s immunization status compared to decisions regarding daily food preparation because the former symbolizes a lack of physical mobility for women, a factor that policymakers must consider when implementing micro-level initiatives to improve women’s autonomy and children’s health [25]. In addition, including dimensions of women’s autonomy may tap into socioeconomic and demographic characteristics that are specific to a culture or social context. Without including additional analyses of specific elements of women’s household autonomy, explanations for its effects on health outcomes are limited.

## Methods

### Data

Data come from the 2011 EDHS. The 2011 EDHS has complete interviews from 16,702 households; 16,515 women aged 15–49 years; and 14,110 men aged 15–59 years. This study’s sample was limited to the 2941 children aged 12–30 months of married women aged 15–49 years in the survey. The child age limits were based on WHO recommendations that children be fully immunized by 12 months of age. There is a 30-month upper limit to cover the cases where children were somewhat late in getting all the vaccines.

### Dependent variable

This analysis focuses on children’s immunization status, an important health indicator because it is one of the earliest ways parents can prevent common childhood diseases/infections. The EDHS collected information on children’s immunizations from vaccination cards and mothers’ verbal responses. The vaccination cards represent routine vaccines whereas maternal recall encompasses both routine vaccines and those done through immunization campaigns. Immunization status was coded as a multi-response categorical variable based on WHO standards: a value of “0” was assigned to children who had received no vaccines, a value of “1” was assigned to children who were partially vaccinated (had received one to seven WHO-recommended vaccines), and a value of “2” was assigned to children who were fully vaccinated (received all eight vaccines).

### Independent variables

The primary independent variable, women’s household autonomy, was gauged through EDHS questions asking women about their household decision-making abilities: “Who usually makes the final decision on the purchase of major household goods; visits to family/relatives; women’s earnings; and women’s own health care?” Responses to each of these questions were coded into three categories: the woman made the sole decision, the woman made the decision jointly with her husband/partner, or the husband/partner made the sole decision. The household autonomy index reflects the number of decisions in which a woman participated alone or jointly with her husband. Specifically, it was created by summing up the total number of decisions in which a woman participated alone or jointly with her husband and ranged in all four scenarios. It ranged from 0 to 4: a low score on the household autonomy index indicates a lower level of household autonomy whereas a high score on the autonomy index indicates a higher level of household autonomy.

I included two dichotomous measures of women’s health access: women who delivered their last child in a hospital/clinic/health care facility versus women who delivered at home and women who received some form of antenatal care for their latest pregnancy versus women who received no antenatal care.

The analysis also included measures of mothers’ and fathers’ socioeconomic status. Mothers’ educational attainment was measured in three categories due to small cell counts at the higher levels of education: no education, primary school education, and secondary school and higher. Fathers’ educational attainment was measured in four categories: no education, primary school education, secondary school education, and higher. Women’s occupation was coded into four categories: no occupation, manual, agriculture, and professional sector. Women’s household wealth status was measured as an index constructed by the EDHS: poorest, poorer, middle, richer, richest. Other control variables included women’s religion, age in years, polygamous marriage, age at first marriage, child’s age, child sex, number of children under 5 years of age in the household, region, and urban/rural residential location.

### Methods

I employed ordinal logistic regressions for the multivariate analyses. “Partially immunized” and “fully immunized” statuses were contrasted against the reference category of “not vaccinated” children. Sample weights were used to make the results nationally representative.

### Ethical clearance

This study is based on an analysis of survey data with all identifying information removed. The 2011 EDHS was approved by the Ethiopia Health and Nutrition Research Institute (EHNRI) Review Board, the National Research Ethics Review Committee (NRERC) at the Ministry of Science and Technology, the Institutional Review Board of ICF, and the CDC. All study participants gave informed consent before participation, and all information was collected confidentially. Use of this data was granted by ICF International.

## Results

### Descriptive statistics

Table 1 presents weighted descriptive statistics. About 15 % of Ethiopian children did not receive any vaccine, though most children were partially vaccinated. Only 24 % of children were fully immunized, meaning that they received all eight doses of vaccines specified by the WHO. The household autonomy index mean of 3.1 means that on average, most women made about three out of four decisions alone or jointly. More specifically, individual decision-making showed that across all scenarios, most women made decisions jointly with their husband/partner rather than solely. Approximately 89 % of women delivered their last child at home, and only 36 % of women received some form of antenatal care. Finally, about 68 % of mothers did not receive a formal education, 47 % of mothers were not in the labor force, and households were evenly spread across the wealth index.

**Table 1:** Women’s reports of autonomy, socioeconomic status, and their children’s immunization status in Ethiopia (weighted data)

	*N* (percent %)	CI
Immunization status		
No vaccinations (reference category)	521 (14.9)	(11.9, 18.5)
Partially vaccinated	1588 (61.0)	(57.4, 64.5)
Fully vaccinated	832 (24.1)	(21.3, 27.1)
Mean number of vaccinations	4.7 (mean)	(4.4, 5.0)
Autonomy index (0–4)	3.1 (mean)	(3.0, 3.2)
Household decision-making		
Person who makes final decision on household purchases		
Mother	185 (4.3)	(3.3, 5.6)
Joint	1647 (61.3)	(58.0, 64.5)
Husband/partner (reference category)	1109 (34.4)	(31.3, 37.7)
Person who makes final decision on visits to family/friends		
Mother	594 (14.4)	(12.5, 16.5)
Joint	1550 (62.0)	(58.8, 65.1)
Husband/partner (reference category)	797 (23.6)	(21.2, 26.2)
Person who makes final decisions regarding respondent’s earnings		
Mother	294 (9.2)	(7.6, 11.2)
Joint	2555 (87.4)	(85.3, 89.3)
Husband/partner (reference category)	92 (3.3)	(2.3, 4.7)
Person who makes final decisions on respondent’s health care		
Mother	453 (12.0)	(10.3, 13.9)
Joint	1633 (59.8)	(56.5, 63.0)
Husband/partner (reference category)	855 (28.2)	(25.3, 31.3)
Health care		
Home delivery	2495 (89.8)	(87.8, 91.4)
Antenatal care	1139 (36.0)	(33.0, 39.1)
Socioeconomic status		
Mothers’ individual level of educational attainment		
No education (reference category)	2040 (68.8)	(65.5, 71.9)
Primary school	740 (26.9)	(22.1, 27.9)
Secondary school and higher	161 (4.4)	(5.1, 7.9)
Fathers’ individual level of educational attainment		
No education (reference category)	1508 (51.0)	(47.4–54.6)
Primary school	1071 (39.6)	(36.3–43.1)
Secondary school	189 (4.9)	(3.7–6.4)
Higher	173 (4.5)	(3.3–6.0)
Women’s occupation		
No occupation (reference category)	1645 (47.2)	(43.7, 50.7)
Manual	215 (7.2)	(5.6, 9.2)
Agriculture	586 (26.9)	(23.8, 30.3)
Professional	495 (18.7)	(16.0, 21.8)
Household wealth		
Poorest (reference category)	904 (23.0)	(19.8, 26.5)
Poorer	513 (22.1)	(19.7, 24.7)
Middle	474 (20.9)	(18.6, 23.4)
Richer	482 (19.3)	(16.5, 22.5)
Richest	568 (14.7)	(12.7, 16.8)

Source: 2011 EDHS; *N* = 2941

Table 2 presents ordinal logistic regression results testing the association between women’s household autonomy and Ethiopian children’s immunization status. Results from this table confirm the importance of women’s household autonomy for child health: net of socioeconomic status, women’s household autonomy was a significant predictor of children’s immunization status. Beginning with the baseline model that only included the household autonomy index, every one unit increase in women’s household autonomy was associated with an expected 16 % increase in the odds of a child being partially immunized compared to the reference category of a child having no vaccines. In model 2, I included health access measures, which slightly diminished the household autonomy effect. Relative to women who did not receive antenatal care, women who received antenatal care had increased odds of children being partially immunized compared to having no vaccines. The inclusion of the socioeconomic variables in model 3 did not dampen the household autonomy effect: net of health care access, education, occupation, and household wealth, increases in women’s household autonomy continued to have significant and positive effect on the odds of children being partially immunized versus not vaccinated at all.

**Table 2:** Odds ratios from ordinal logistic regressions predicting Ethiopian children’s immunization status^ae^

	Partially immunized (vs. no vaccines)			Fully immunized (vs. no vaccines)		
	Model 1	Model 2	Model 3	Model 1	Model 2	Model 3
Autonomy index	1.16*	1.13****	1.16**	1.36**	1.29**	1.32**
	(1.00, 1.35)	(0.98, 1.31)	(1.00, 1.33)	(1.13, 1.63)	(1.07, 1.56)	(1.10, 1.590)
Delivered at home		0.91	1.04		0.61	0.80
		(0.33, 2.53)	(0.39, 2.81)		(0.20, 1.88)	(0.27, 2.35)
Antenatal care		2.62***	2.45***		5.68***	4.95***
		(1.57, 4.38)	(1.46, 4.11)		(3.30, 9.79)	(2.89, 8.50)
Mothers’ education^b^						
Primary school			1.76*			2.32**
			(1.09, 2.86)			(1.64, 4.01)
Secondary school and higher			2.31			3.72
			(0.44, 12.17)			(0.76, 18.20)
Fathers’ education^b^						
Primary school			1.17			0.96
			(0.75, 1.82)			(0.58, 1.58)
Secondary school			0.98			0.73
			(0.34, 2.77)			(0.22, 2.36)
Higher			1.08			0.89
			(0.34, 3.39)			(0.25, 3.13)
Occupation^c^						
Manual			0.43*			0.43****
			(0.20, 0.95)			(0.16, 1.13)
Agriculture			1.28			1.57
			(0.82, 2.01)			(0.91, 2.73)
Professional			1.09			1.04
			(0.67, 1.76)			(0.61, 1.80)
Wealth index^d^						
Poorer			0.69			1.00
			(0.42, 1.13)			(0.53, 1.88)
Middle			0.65****			0.75
			(0.40–1.04)			(0.41, 1.36)
Richer			0.5*			0.99
			(0.32, 0.95)			(0.50, 1.94)
Richest			1.09			2.95****
			(0.341, 2.90)			(0.98, 8.81)
Constant	3.18	1.97	0.92	3.36	1.48	0.37
	(0.51, 19.72)	(0.32, 12.06)	(0.172, 4.92)	(0.46, 24.85)	(0.21, 10.63)	(0.06, 2.28)
Observations	2941	2941	2941	2941	2941	2941

*p < 0.05; **p < 0.01; ***p < 0.001; ****p < 0.10

^a^Each model controls for women’s religion, mother’s age in years, polygamous marriage, age at first marriage, child’s age, child sex, number of children under 5 years of age in the household, region, and urban/rural residential location

^b^Reference category is no education

^c^Reference category is no occupation

^d^Reference category is poorest

^e^Confidence interval in parentheses

Source: 2011 Ethiopia Demographic and Health Surveys

The second half of the table displays results predicting full immunization status versus no vaccines. Model 1 shows that a one-unit increase in women’s household autonomy is related to a 36 % increase in the odds of a child being fully immunized versus not having any vaccines. In model 2, the household autonomy index odds ratio is reduced in size yet remained highly statistically significant, net of health access. It should be noted that compared to women who received no antenatal care, women who received antenatal care had higher odds of children being fully immunized versus having no vaccines. The full array of socioeconomic-status variables in model 3 did nothing to diminish the statistical strength of women’s household autonomy.

Table 3 shows the multinomial logistic regression results modeling the relationships between individual measures of women’s household autonomy and immunization status. Looking across all the models, only decisions related to finances had a significant effect on partial immunization status. In the first full model, for example, compared to women who said their husband made the final decision on their earnings, women who said they made joint decisions about their earnings had 4.3 times higher odds of their children being partially immunized versus having no vaccines, net of all control variables. In the second full model, joint decisions on earnings (compared to husbands making the sole earnings decision) increased the odds of children being fully immunized by 8.1 times versus not being vaccinated.

**Table 3:** Odds ratios from ordinal logistic regressions predicting Ethiopian children’s immunization status using individual measures of women’s autonomy^ae^

	Partially immunized (vs. no vaccines)			Fully immunized (vs. no vaccines)		
	Model 1	Model 2	Model 3	Model 1	Model 2	Model 3
Individual measures of women’s autonomy^f^						
Person who makes final decision on household purchases						
Mother	2.11	1.92	1.86	2.12	1.68	1.67
	(0.84, 5.31)	(0.74, 4.99)	(0.74, 4.71)	(0.70, 6.44)	(0.52, 5.39)	(0.452, 5.32)
Joint	1.18	1.07	1.10	1.30	1.07	1.09
	(0.78, 1.78)	(0.70, 1.63)	(0.72, 1.66)	(0.80, 2.11)	(0.65, 1.75)	(0.66, 1.77)
Person who makes final decision on visits to family/friends						
Mother	1.15	1.01	1.00	1.55	1.28	1.26
	(0.66, 2.03)	(0.56, 1.82)	(0.55, 1.81)	(0.73, 3.28)	(0.60, 2.72)	(0.58, 2.72)
Joint	0.97	0.94	0.97	1.19	1.19	1.23
	(0.61, 1.53)	(0.59, 1.50)	(0.60, 1.55)	(0.70, 2.05)	(0.69, 2.05)	(0.71, 2.13)
Person who makes final decisions regarding respondent’s earnings						
Mother	2.78****	2.78	3.38****	4.63*	4.96*	6.53**
	(0.87, 8.89)	(0.82, 9.40)	(1.00, 1.42)	(1.34, 15.98)	(1.22, 20.18)	(1.68, 25.54)
Joint	3.16**	3.57**	4.29**	4.71**	6.30***	8.13***
	(1.34, 7.49)	(1.53, 8.35)	(1.69, 10.89)	(1.86, 11.89)	(2.27, 17.54)	(2.59, 25.34)
Person who makes final decisions on respondent’s health care						
Mother	1.40	1.47	1.42	1.91	2.00****	1.79
	(0.70, 2.81)	(0.73, 2.98)	(0.68, 2.93)	(0.87, 4.17)	(0.90, 4.41)	(0.79, 4.10)
Joint	1.08	1.10	1.06	1.22	1.28	1.19
	(0.71, 1.64)	(0.72, 1.70)	(0.67, 1.67)	(0.71, 2.09)	(0.74, 2.22)	(0.67, 2.12)
Health care						
Delivered at home		0.87	0.96		0.58	0.74
		(0.31, 2.45)	(0.37, 2.50)		(0.19, 1.81)	(0.26, 2.13)
Antenatal care		2.75***	2.54***		6.04***	5.08***
		(1.64, 4.60)	(1.54, 4.18)		(3.51, 10.39)	(2.98, 8.65)
Mothers education^b^						
Primary school			1.78*			2.30**
			(1.08, 2.94)			(1.31, 4.04)
Complete secondary school and higher			2.28			3.66****
			(0.47, 11.11)			(0.83, 16.27)
Fathers’ education^b^						
Primary school			1.10			0.91
			(0.72, 1.68)			(0.55, 1.46)
Secondary school			1.05			0.80
			(0.35, 3.10)			(0.24, 2.67)
Higher			1.07			0.87
			(0.35, 3.31)			(0.25, 2.98)
Occupation^c^						
Manual occupation			0.56			0.57
			(0.26, 1.20)			(0.22, 1.50)
Agriculture occupation			1.34			1.64****
			(0.85, 2.11)			(0.93, 2.89)
Professional occupation			1.37			1.31
			(0.77, 2.40)			(0.69, 2.50)
Wealth index^d^						
Poorer			0.74			1.06
			(0.43, 1.27)			(0.55, 2.06)
Middle			0.66****			0.74
			(0.41, 1.06)			(0.40, 1.37)
Richer			0.52*			0.91
			(0.31, 0.87)			(0.47, 1.78)
Richest			1.09			2.87****
			(0.42, 2.85)			(0.97, 8.46)
Constant	1.40	0.78	0.27	1.08	0.37	0.69
	(0.21, 9.18)	(0.12, 5.15)	(0.04, 1.69)	(0.14, 8.33)	(0.05, 2.92)	(0.01, 0.45)
Observations	2941	2941	2941	2941	2941	2941

*p < 0.05; **p < 0.01; ***p < 0.001; ****p < 0.10

^a^Each model controls for women’s religion, mother’s age in years, polygamous marriage, age at first marriage, child’s age, child sex, number of children under 5 years of age in the household, region, and urban/rural residential location

^b^Reference category is no education

^c^Reference category is no occupation

^d^Reference category is poorest

^e^Confidence interval in parentheses

^f^Reference group is husband makes final decision

Source: 2011 Ethiopia Demographic and Health Surveys

Additional models analyzing the relationship between women’s household autonomy and children’s number of vaccines using negative binomial models (results not presented) showed a positive relationship: net of socioeconomic status and health access, 1-unit increases in women’s household autonomy were related to 0.06-unit increases in the number of vaccines.

## Discussion

Several studies have showed how women’s autonomy is positively related to children’s health outcomes [9, 14, 26], yet few have looked at how household autonomy is related to children’s immunization status [22]. This study is built upon these prior analyses and tested the relationship between women’s household autonomy and Ethiopian children’s immunization status. The first key finding from this study was the importance of women’s household autonomy for children’s immunization status. This pattern is similar to results observed by Singh et al. [22] in Nigeria, where women’s autonomy was also positively correlated with children’s immunization status. Including mothers’ and fathers’ individual-level educational attainment, household wealth, and occupation did not dampen the robust and significant relationship between women’s overall participation in decisions related to their movement, household purchases, earnings, use of antenatal care, and their children’s immunization status. Women’s socioeconomic status has been identified as a key means of improving household autonomy and gender equity: more educated women are more likely to work outside the home in better paying occupations and have better access to health services and health-related knowledge than less educated women [27]. The results of this study show that for Ethiopian women, education was not a substitute for autonomy. That is, Ethiopian women’s household autonomy is not solely created through access to education but through greater freedom to make decisions concerning their livelihoods. Therefore, policymakers should not only continue to provide Ethiopian women with safe and accessible schools but should also implement gender-related initiatives that specifically target women’s autonomy within the home. Yet it should be noted that women’s household autonomy was not as important to children’s immunization as women’s use of antenatal care. As suggested by Jennings et al. [28], women’s household autonomy and antenatal care use might not be individually exclusive pathways for improving children’s health. That is, women who make sole or joint decisions with their spouse or partner might be more likely to interpret antenatal care as either a “shared domain” or more likely to solicit antenatal care compared to women with low household autonomy [28–30].

A second key finding was the significance of individual measures of women’s household autonomy. More specifically, women who had decision-making abilities related to financial allotments were more likely to have their child partially or fully immunized, compared to women who had no part in these decisions. Women with the freedom to make financial decisions means that they can decide to use their resources to pay for preventative health services for their children. More interesting is the result that women who made financial decisions jointly with their husband were even more likely to get their children vaccinated compared with women who made financial decisions independently. This finding may mean that joint decision-making represents cohesiveness between wives and husbands and an active way that men can improve children’s health outcomes that is not necessarily related to socioeconomic status. Though many gender initiatives are typically female-focused, this result suggests that policymakers consider how initiatives that focus on improving marital communication and equity can both improve a sense of spousal togetherness and improvement in children’s health outcomes.

There are some limitations to this study. First, the cross-sectional nature of the EDHS means that causal relationships between individual measures of women’s household autonomy and children’s immunization status cannot be established with certainty. Second, the EDHS does not ask questions about women’s role within society as a whole, which might be related to children’s immunization status. It is important for future research on women’s autonomy to capture both household and social measures of women’s autonomy and to use longitudinal data to understand its long-term impact on children’s health across the life course.

## Conclusion

Research on immunization coverage is not only an important indicator of health care utilization but also a measureable indicator of the progress toward the 2015 MDG. Understanding factors that could assist women in seeking health care for their children is also important in advancing the MDG’s gender-related initiatives. This study provides important insight into one of the many ways that empowering women shapes the lives of those around them.

## Abbreviations

DHS: Demographic Health Surveys
